# Lower limb exoskeletons for older adults: key assistance parameters and evaluation of ankle assistive strategies via musculoskeletal modelling

**DOI:** 10.3389/fspor.2026.1888675

**Published:** 2026-08-28

**Authors:** Josée Mallah, Luigi G. Occhipinti

**Affiliations:** 1 Electrical Engineering Division, Department of Engineering, University of Cambridge, Cambridge, United Kingdom; 2 Wolfson School of Mechanical, Electrical and Manufacturing Engineering, Loughborough University, Loughborough, United Kingdom

**Keywords:** aging, biomechanics, exoskeletons, gait analysis, musculoskeletal modelling

## Abstract

Aging comes with physiological changes resulting in alterations to gait, decline of muscle function, and balance that contribute to reduced mobility, increased metabolic cost of walking, and elevated fall and injury risks in older adults. Wearable lower-limb exoskeletons have emerged as a promising strategy to support healthy aging; however, identifying optimal assistance strategies remains challenging due to the complexity and cost of experimental testing. In this study, we combined musculoskeletal modelling and simulations to identify key assistance parameters for older-adult gait and evaluate different ankle exoskeleton configurations computationally, based on muscle activation, metabolic consumption, device weight and power requirement, efficiency, and performance. Motion capture, force plate, and electromyography data from younger and older adults were processed using OpenSim-based musculoskeletal simulations with age-adjusted muscle parameters. Age-related gait differences were quantified across spatiotemporal, kinematic, kinetic, and muscle activation metrics. Subsequently, unilateral and bilateral ankle assistance strategies providing plantarflexion (PF), dorsiflexion (DF), or combined plantarflexion–dorsiflexion (PFDF) support were simulated using unidirectional path actuators representative of cable-driven exoskeletons. Older adults exhibited reduced ankle and hip range of motion, decreased plantarflexion, delayed joint moments, shorter step length, and increased double support time compared with younger adults. These changes suggest that elderly gait can be improved with kinematic assistance at the ankles and hips, timed kinetic assistance at the knees and hips, as well as balance support, hence an opportunity for exoskeleton intervention. Simulated ankle assistance reduced muscle activations and metabolic expenditure, with bilateral PFDF assistance providing the greatest overall benefits. While dorsiflexion-only assistance reduced power requirements, it occasionally increased metabolic consumption. Overall, bilateral assistance outperformed unilateral configurations across most performance metrics. These findings provide biomechanical design guidelines for future lower-limb exoskeletons targeting mobility enhancement and fall-risk reduction in aging populations and demonstrate the value of computational biomechanics for screening assistive-device configurations prior to experimental implementation.

## 1 Introduction

The number and proportion of older adults aged 60 years or more is on the rise—WHO is predicting an increase from 1 billion people in 2019 to 1.4 billion by 2030 and 2.1 billion by 2050 (1). Aging comes with a weakening of the body, reduction of the muscle force, and difficulties with motor control resulting from changes in the central and peripheral nervous systems. Thus, the person's mobility is affected, which is in turn associated with secondary health issues, such as blood pressure, diabetes, cardiovascular disease, and mortality (2–5). Balance issues are common, with 13% of adults aged 65%–69% and 35% of adults aged 85 and older self-reporting imbalance (6). Falling is therefore a direct consequence, with an estimated annual fall prevalence of 28% in people aged 65 and above (6, 7); it is the main cause of traumatic injuries in adults over 65 years of age, with most of these incidents happening during walking (3, 5, 8, 9). Aging is also associated with a higher metabolic cost of walking, i.e., increased caloric expenditure and reduced energy efficiency (3, 10–13).

Aging particularly affects muscular behaviour leading to the adoption of different muscle strategies during gait compared to younger people (14). Changes in muscle mechanisms, resulting from muscle atrophy and remodelling (15), include a substantial loss of strength (16–18), prolonged twitch contractions (17, 19), increased passive stiffness (20, 21), and slowing of the rate of muscle force development (22, 23).

The cutoff age of 60 years was frequently used in the literature to distinguish between older and younger adults (14). While gait stability starts decreasing in the 40–50s (6, 24), the gait pattern still undergoes changes beyond the age of 60 (14, 25), as (26) found that 25% of people aged 70–74 had gait changes vs. nearly 60% of people aged 80–84. Therefore, in order to conduct a comprehensive gait comparison between younger and older adults, distant age groups better be considered, with the younger category being under 35–40 years of age and the older category being 70 years and older.

Recently, there has been an increased interest in providing assistance to older adults and helping them carry out their daily activities, stay active, and live independently with a good quality of life, especially under the title of “healthy aging” (27, 28). Thus, efforts are being targeted towards the development of assistive technologies such as exoskeletons (29), as traditional assistive devices fail to successfully meet all the requirements. Simple aids, like sticks and crutches, can provide short-term or low-level support, and fail when a higher-level support is needed. Wheelchairs and scooters can solve this problem but are bulky and impractical in small spaces like homes; wheelchair users find themselves needing to install chair lift and ramps and change fittings etc. which require extra financial costs despite the low price of the wheelchair or scooter itself. All these issues are resolved by wearable, body-fitting exoskeletons, which require minimal lifestyle changes while allowing the user to stay active and independent (30–32).

Exoskeletons normally aim to restore natural gait, i.e., that of a healthy young person, often either by tracking set desired joint angles or supplementing the joint with extra torque (33), which we refer to here as kinematic and kinetic assistance, respectively. Moreover, exoskeleton controls can be optimised for different purposes, such as to assist with balance (34, 35), and reduce muscle force (36).

In particular, we are interested in devices incorporating active mechanical elements and providing additional power (37), as passive devices, despite their lightweight, have limited controllability and struggle to break the metabolic consumption barrier, due to their inability to provide enough positive power to overcome the added device mass (38–40). Ankle exoskeletons are promising for elderly assistance, as the ankle joint plays an essential role in human gait, producing more mechanical power than either the knee or the hip and supporting balance during walking (41–43). Assisting the ankle joint can provide optimal metabolic consumption savings (44), as increasing the mechanical power output of the ankle decreases the power demands on the hip, leading to improved walking efficiency (43).

Many actuation techniques can be applied in ankle exoskeletons, including pneumatic, compliant (e.g., series-elastic or variable stiffness), and direct-drive (DDA) actuators (33). Under DDA, and while electric motors are often directly coupled to the outputs, they are frequently combined with ropes and Bowden cables for power transmission either (1) from off-body actuator units to the wearable module in tethered exoskeletons (45, 46), or, most interestingly, (2) from actuators worn higher up on the body (e.g., back, waist, or calf) to the ankle module, hence providing a soft, body-conforming actuation in wearable exoskeletons (46–49), as heavy actuators are better placed near the body center of mass instead of distally to minimize the extra metabolic expenditure associated with their added weight (50, 51). However, only a small part of the cable closest to the ankle is active, i.e., can be pulled and shortened.

Ankle exoskeletons can be worn unilaterally (46, 52) or bilaterally (45, 53). Hence, our hypothesis is that bilateral assistance provides greater benefits for older adults as aging affects the whole body equally, in contrast to other motor problems stemming from neurological conditions such as stroke which normally affects only one side of the body. An exoskeleton can be actuated in up to 6 degrees of freedom (54), but most commonly, assistance is provided for either plantarflexion (PF) (55, 56), dorsiflexion (DF) (57, 58), or both (PFDF) (59, 60); hence, we would like to test which angle would provide greater benefits for older adults when assisted.

The evaluation of exoskeleton design and their benefits through experimental trials is inefficient (61) in terms of time and cost, as testing different designs requires manufacturing different devices and conducting human trials. Therefore, musculoskeletal simulation software can be used as a cost-effective computational tool. Even though accurate predictions of the effects of assistive devices in simulation is quite challenging, especially in terms of accounting for device mass, ideal, massless devices can be used to provide lossless transmission of torque to the limbs, without having torque or power limits, which is useful for comparing different types of devices on multiple subjects (61). This framework has been successfully used previously to simulate ankle, knee, and hip assistive devices during running (62), heavy weight carriage (61), and even elderly assistance (63). However, these studies used coordinate actuators acting which act directly on the joint and are able to provide bidirectional torque. However, these actuators do not model cable-driven actuation accurately, especially that a cable can normally only be pulled/shortened and hence providing only unidirectional power.

In this paper, we suggest that walking assistance for older adults can possibly be achieved by restoring their gait biomechanics to those of healthy young adults. Hence, we analyse the gait of older adults and compare it with that of younger adults, to observe the effect of aging on spatiotemporal, kinematic, and kinetic gait parameters as well as muscle activations, and prescribe exoskeleton assistance corresponding to these parameters, in terms of improving balance, as well as the range of joint angles and torques, and reducing muscle force, respectively. Then, we use unidirectional path actuators to provide ankle assistance to older adults during gait, in plantarflexion, dorsiflexion, or both, unilaterally and bilaterally, and evaluate the benefits provided.

## 2 Methods

### 2.1 Biomechanical data

The biomechanical data used in this paper are taken from the publicly-available full-body motion capture dataset by Van Criekinge et al. (2023) (64). Twenty able-bodied subjects were considered—10 younger and 10 older adults (Table 1)—being the most anthropometrically similar within the dataset (as younger subjects tend to be taller and fitter compared to their older counterparts). The dataset contains motion capture, force plate, and electromyography (EMG) data recorded on both sides for the following muscles: rectus femoris, vastus lateralis, biceps femoris, semitendinosus, tibialis anterior, gastrocnemius, and erector spinae. The data were collected using a three-dimensional passive motion capture system [8 Vicon T10 cameras, ©Vicon Motion Systems Ltd., Oxford, UK; reflective markers (14mm, ©B&L Engineering, California, USA)], 4 ground embedded force plates (3 AMTI type OR 6–7 force plates, 46 cm × 50 cm × 8 cm, ©Advanced Mechanical Technology, Inc., Watertown USA; and 1 AccuGait® force plate, 50 cm × 50 cm × 4 cm, ©Advanced Mechanical Technology, Inc., Watertown USA) and a synchronized 16-channel telemetric wireless surface EMG system (Zerowire, ©Cometa, Barregio, Italy) with 14 disposable gel electrodes (Covidien KendallTM). Participants walked over a 12-meter walkway at a self-selected speed, without using any walking aids.

**Table 1:** Subject information.

	Subject	Age	Gender	Weight (kg)	Height (cm)
Older group	SUBJ01	86	M	64	158
	SUBJ02	85	F	78	150
	SUBJ05	84	F	50	145
	SUBJ08	82	F	72	160
	SUBJ16	77	M	86	175
	SUBJ19	76	M	79	175
	SUBJ22	74	F	72	158.5
	SUBJ25	73	F	61	159.5
	SUBJ26	72	F	69	160.5
	SUBJ28	72	F	61	158.5
	Mean ± std	78.1 ± 5.3		69.2 ± 10.0	160 ± 8.8
Younger group	SUBJ94	33	F	59	157.5
	SUBJ95	33	F	70	160.5
	SUBJ96	33	M	84	179.5
	SUBJ109	25	F	65	170.5
	SUBJ110	25	F	67	164.5
	SUBJ112	24	M	76	176.5
	SUBJ114	24	M	84	184
	SUBJ115	24	F	56	158
	SUBJ116	21	F	65	159
	SUBJ117	21	F	66	167
	Mean ± std	26.3 ± 4.6		69.2 ± 9.0	167.7 ± 9.1

Raw EMG signals were filtered using a 4th order Butterworth filter with cutoff frequencies of 10 and 300 Hz, rectified, and smoothed with a 50 ms moving average filter to obtain the linear envelope using MATLAB (R2023b, The MathWorks Inc., Massachusetts, USA).

### 2.2 Opensim pipeline

#### 2.2.1 Comparison between young- and old-age gait

OpenSim (65) is the musculoskeletal modelling software used to process motion data, along with the Gait2392 generic model, which has commonly been used in the literature (66–68). It has 23 degrees of freedom and 92 Thelen Hill-type musculotendon actuators representing 76 muscles in the lower limbs and torso. The unmodified model is used for the 10 younger subjects; however, muscle parameters have been modified to reflect the effects of muscle atrophy and remodelling that occur with age according to Thelen's suggestions (15) for the 10 older subjects. Isometric strength is reduced by 30%, which is comparable to changes observed in lower extremity muscles (17), because of muscle atrophy resulting from the decline in the total number and size of muscle fibres (16, 18, 69). The maximum contraction velocity was reduced by 20% from 10 to 8 optimal fibre lengths per second, which is likely due to a preferential loss of fast twitch motor units (17) and fast twitch fibre cross-sectional area (70), and changes of contraction characteristics of some fibre types (71–73).

Passive muscle strain due to maximum isometric force was decreased from 0.6 for younger adults (74) to 0.5 for older adults, as passive muscle tension accounts for a higher proportion of the total tension in old muscles during stretching (75). The deactivation time constant was increased from 50 to 60 ms, as the rate of muscle deactivation is slowed in old muscles (76–78).

**Figure 1:**
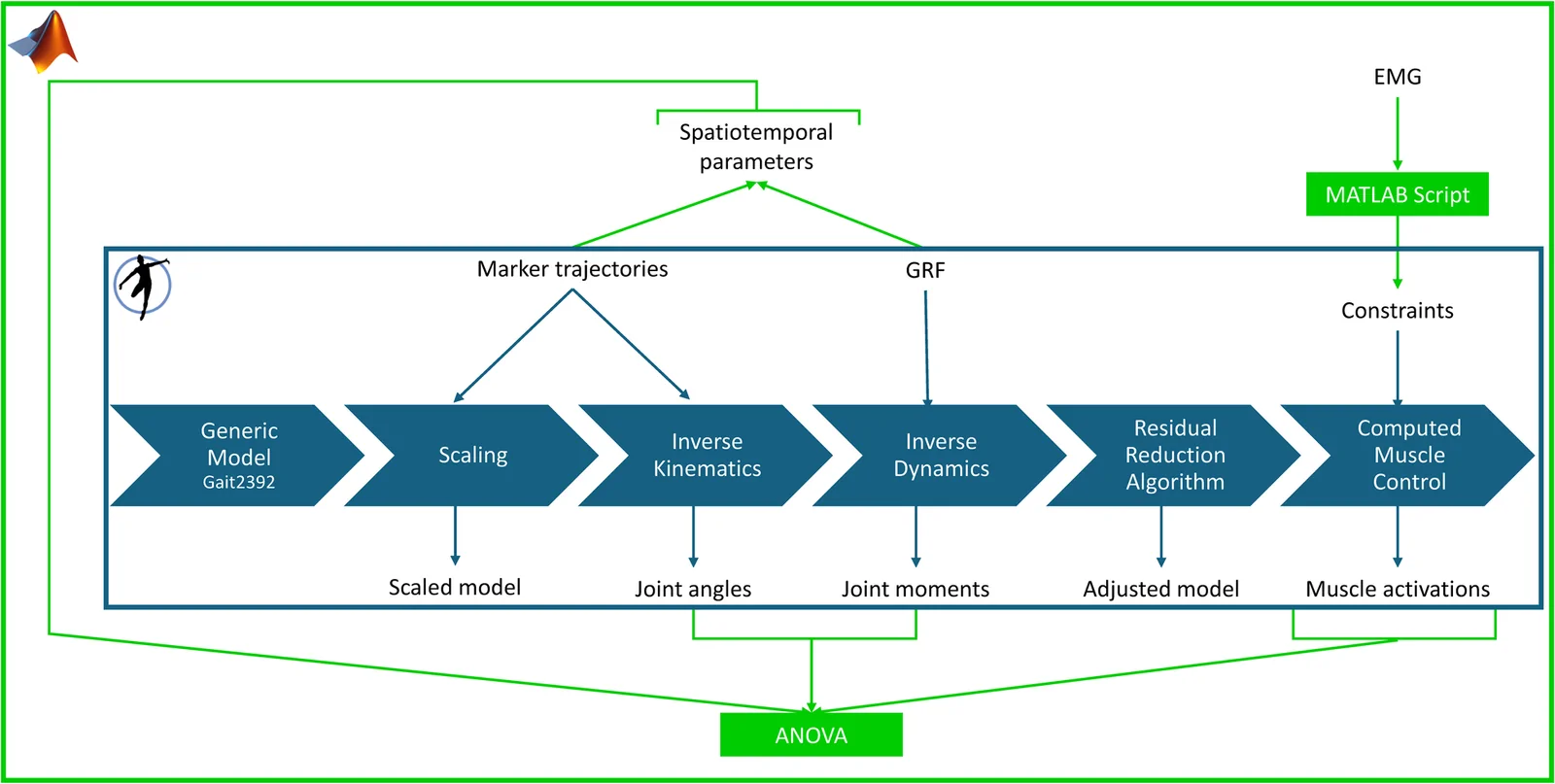
Overview schematic of the simulation pipeline using OpenSim and MATLAB software.

The OpenSim tools were applied in order (Figure 1): inverse kinematics, inverse dynamics, residual reduction algorithm (RRA), and Computed Muscle Control (CMC), which calculates the muscle excitations that drive the model, and provides estimations for all the muscles in the model, i.e., many more than what can be realistically evaluated with EMG. However, the excitations estimated by CMC can fail to exactly reproduce actual EMG data, especially in older adults (79). Hence, a MATLAB script was written to convert each muscle's EMG data into sets of minimum and maximum constraints. The algorithm envelopes the EMG signal by finding the maximum and minimum EMG amplitude in windows of 5 ms and assigns nodes/constraints within which the computed activations are allowed to vary. The script outputs 2 sets of minimum and maximum nodes to be included in the CMC constraints file. Details on the script and a validation of its outcomes are reported in the Supplementary Material.

#### 2.2.2 Simulating ankle assistance

**Figure 2:**
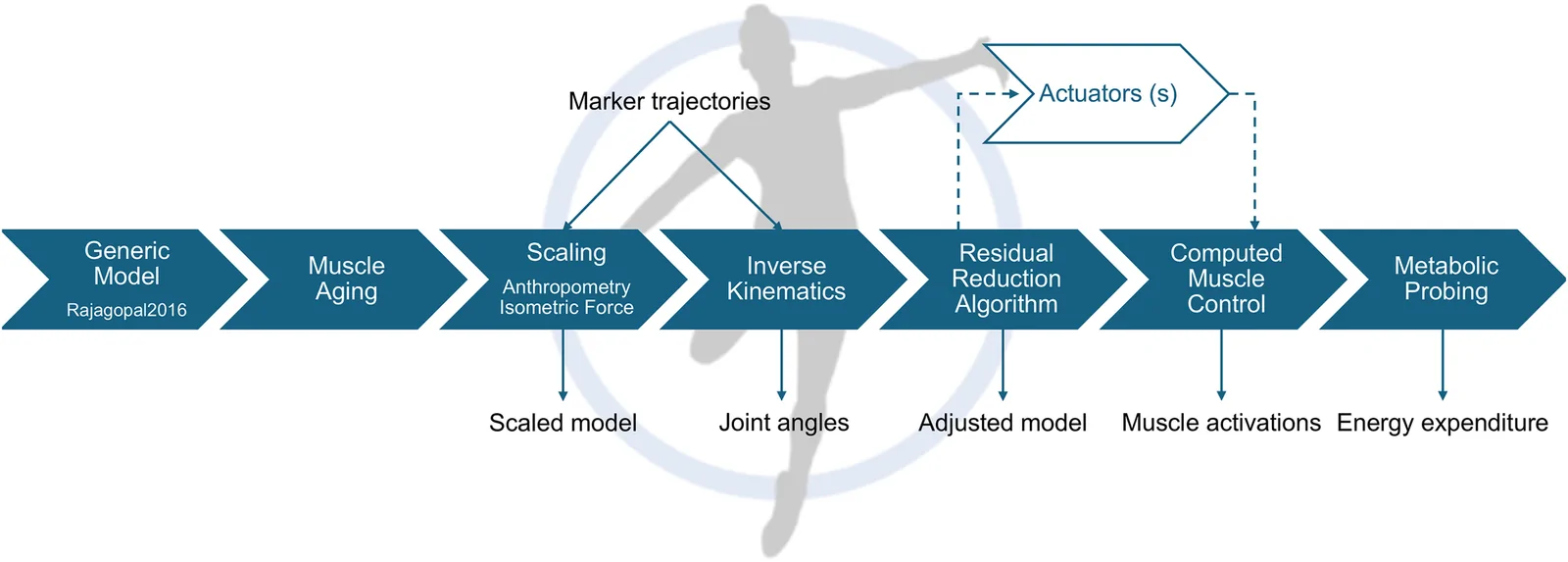
OpenSim pipeline.

OpenSim is used along with the Rajagopal2016 generic model (80). It has 39 degrees of freedom, of which 8 were locked for being irrelevant to our study (ankle eversion, toe flexion, wrist flexion, wrist deviation). The model's muscle parameters were modified to reflect the effects of age-induced muscle atrophy and remodeling as described in Section 2.2.1 above. The model is first scaled to the anthropometric dimensions of each subject using the Scale tool. Then the maximum isometric forces are scaled using a regression equation based on subject mass and height (80–82). The OpenSim pipeline (Figure 2) was then followed, starting from Inverse Kinematics, followed by RRA and the CMC, while using the Umberger2010MuscleMetabolicsProbe (83–85) to solve for metabolic consumption. The CMC tool was run for the unassisted as well as the assisted scenarios. In order to simulate exoskeleton assistance, a unidirectional path actuator that applies force between 2 points is defined between the back of calf and the heel for PF, the front of the calf and the midfoot for DF, while both actuators were used for PFDF assistance. The actuators were added unilaterally and bilaterally.

The CMC tool calculates the muscle excitations that drive the model by solving for the objective function (Equation 1), minimizing the sum of squared muscle activations $a_{i}$ and penalising the forces or moments $f_{j}$ applied by reserve and residual actuators by adjusting the weighing factor $w_{j}$ (corresponding to the optimal force in OpenSim):

$$J=\;\overset{nMuscles}{\underset{i=1}{\sum }}\;a_{i}^{2}+\;\overset{nRes}{\underset{j=1}{\sum }}\;{\left(\frac{f_{j}}{w_{j}}\right)}^{2}$$ (1)

In the assisted scenarios, the CMC also solves for the optimal exoskeleton activation, considered as a residual force. Hence, a high optimal force (equivalent to a moment of 1,000 Nm) is applied to the actuators so that the optimizer prefers using the device over the muscles.

### 2.3 Data analysis

#### 2.3.1 Comparison between young- and old-age gait

Muscle groups are considered to better observe how the obtained changes affect the movement. Group muscle activations are the sum of average activations of its member muscles, averaged for the right and left sides. Statistical analysis of the gait parameters was evaluated using a one-way analysis of variance (ANOVA), performed in MATLAB. Where a significant difference (*p* < 0.05) is present, the relative difference between the means of the older and younger groups is reported, along with the95% confidence interval (CI) for the mean difference, and Hedges' *g* as a standardized effect size (a small-sample corrected version of Cohen's dz). The considered parameters are as follows:

##### 2.3.1.1 Spatiotemporal parameters

Spatiotemporal parameters: stance time, swing time, stride time, stance percentage (relative to full stride), swing percentage, double support time and percentage, step length, stride distance, and speed. One full stride, or gait cycle, is delimited by two consecutive heel strikes of the same foot, i.e., when the foot hits the force plate (20 N threshold). However, the available GRF data only records one heel strike per foot. Thus, the following heel strike was identified when the heel marker velocity reaches the same value again. Stance is the time between heel strike and toe-off, during which the foot is in contact with the ground, while swing is the remaining part of the stride. Double support is the time during which both feet are in contact with the ground, which occurs at the beginning and end of the stance phase. However, as we only have GRF data for 2 steps, the first half of the double support phase (beginning of the stride) cannot be determined, and hence the values reported here are only for the second half of double support during late stance. Step length is the difference between the right and left heel marker positions in the sagittal plane at heel strike. Stride distance is the difference between the same heel marker position at the consecutive heel strikes in the sagittal plane. Walking speed was then calculated by dividing stride distance by stride time.

##### 2.3.1.2 Kinematic parameters

Kinematic parameters: the range of motion (ROM) for the ankle, knee, and hip joints, as well as the maximum angles in the sagittal plane [plantarflexion (−)/dorsiflexion (+), knee flexion (−)/extension (+), and hip extension (−)/flexion (+)].

##### 2.3.1.3 Kinetic parameters

Kinetic parameters: the peak moment values and times (as percentage of the gait cycle) for the ankle, knee, and hip joints.

##### 2.3.1.4 Muscle activations (OpenSim estimates)

The average activation and numerical integration over time are computed for each muscle during 15%–100% stride. The total activation (sum of average or integrated activations) is calculated for each muscle group, and then the right and left sides are averaged.

#### 2.3.2 Simulating ankle assistance

A set of parameters were calculated from the results to evaluate the performance of the devices. In order to assess the impact on the muscles, we compute the numerical integration of the sum of muscle forces over the gait cycle (namely 15%–100%), and then the percent reduction compared to the unassisted scenario (musc%). We calculate the gross average metabolic rate by performing a numerical integration of the energy consumption of the whole body (including basal rate), and the percent reduction compared to the unassisted value (ga_metab_reduc). The gross average metabolic rate is normalized by subject mass and walking speed, yielding the cost of transport (COT). Then, we find the absolute reduction of metabolic rate compared to the unassisted scenario, and normalize it by subject mass (abs_ga_metab_reduc). The peak positive power is the maximum over time of the sum of positive power of the actuator(s); it helps evaluate the cost of carrying a device, as peak power can be used to estimate the actuator mass, one of the heaviest components in an exoskeleton (51, 86). We also calculate the average positive device power, and normalize it by subject mass, as it can be used to estimate the battery life of untethered devices. All powers are normalized by the subject weight. An efficiency metric is computed as the ratio of the absolute reduction in metabolic rate to the peak positive device power, preferring devices that provide higher metabolic savings with less power requirements, along with a performance indicator, calculated as the ratio of the absolute reduction of metabolic rate to the average positive device power (both unitless metrics) (61).

Then we want to compare between (1) the 3 directions of bilateral assistance (PF, DF, PFDF), (2) the 6 modes of unilateral assistance (PFr, PFl, DFr, DFl, PFDFr, PFDFl; r/l corresponds to right/left), and (3) the 3 unilateral and bilateral assistance modes for each assistance direction (PFr, PFl, PF; DFr, DFl, DF; PFDFr, PFDFl, PFDF). Normality was assessed using Shapiro–Wilk tests for each condition, and Repeated-measures ANOVA was used when no more than one condition violated normality. Otherwise, the Friedman test was used instead. Sphericity was assessed using Mauchly's test; when sphericity was violated, Greenhouse-Geisser corrected *p*-values were reported. Bonferroni-corrected paired comparisons were used following repeated-measures ANOVA, whereas Dunn–Šidák corrected comparisons were used following Friedman tests. Significance level is set at *p* < 0.05. For pairwise *post-hoc* comparisons, we report 95% confidence intervals and Cohen's dz as the standardized paired effect size; hence, confidence intervals that did not include zero were interpreted as indicating a consistent direction of effect, while Cohen's dz values were used to describe the magnitude of pairwise effects.

All data analysis was performed in MATLAB (R2024b, The MathWorks Inc., Massachusetts, USA).

## 3 Results

### 3.1 Key assistance parameters

#### 3.1.1 Spatiotemporal parameters

The results are shown in Table 2. While no significant difference in stance time was observed between both the younger and older groups, swing time was on average 8.35% shorter for the older group (*F* = 11.44, *p* = 0.0033, 95% CI = [−0.06, −0.01], *g* = −1.45), which is equivalent to a longer stance percentage (+3.02%) and lower swing percentage relative to a full stride (*F* = 13.42, *p* = 0.0018, 95% CI = [0.78, 2.86], *g* = 1.57). The older group exhibited 10.63% higher double support percentages (*F* = 5.76, *p* = 0.0274, 95% CI = [0.14, 2.09], *g* = 1.03).

**Table 2:** Average ± standard deviation of the 10 considered spatiotemporal parameters for both the young and older groups.

Spatiotemporal parameter	Young group	Older group
Stance time (s)	0.65 ± 0.06	0.64 ± 0.05
Swing time (s)	0.43 ± 0.03	0.39 ± 0.02
Stride time (s)	1.08 ± 0.08	1.04 ± 0.06
Stance percentage (%)	60.27 ± 0.96	62.08 ± 1.24
Swing percentage (%)	39.73 ± 0.96	37.92 ± 1.24
Double support time (s)	0.11 ± 0.02	0.12 ± 0.02
Double support percentage (%)	10.48 ± 1.05	11.59 ± 1.02
Step Length (m)	0.60 ± 0.06	0.53 ± 0.07
Stride distance (m)	1.27 ± 0.10	1.13 ± 0.13
Speed (m/s)	1.18 ± 0.10	1.09 ± 0.13

Older adults have an 11.92% shorter step length (*F* = 6.99, *p* = 0.0165, 95% CI = [−0.13, −0.01], *g* = −1.13) and an 11.06% shorter stride distance (*F* = 7.31, *p* = 0.0145, 95% CI = [−0.25, −0.03], *g* = −1.16. No statistically significant difference in walking speed was observed (*F* = 2.83, *p* > 0.1).

#### 3.1.2 Kinematic parameters

**Figure 3:**
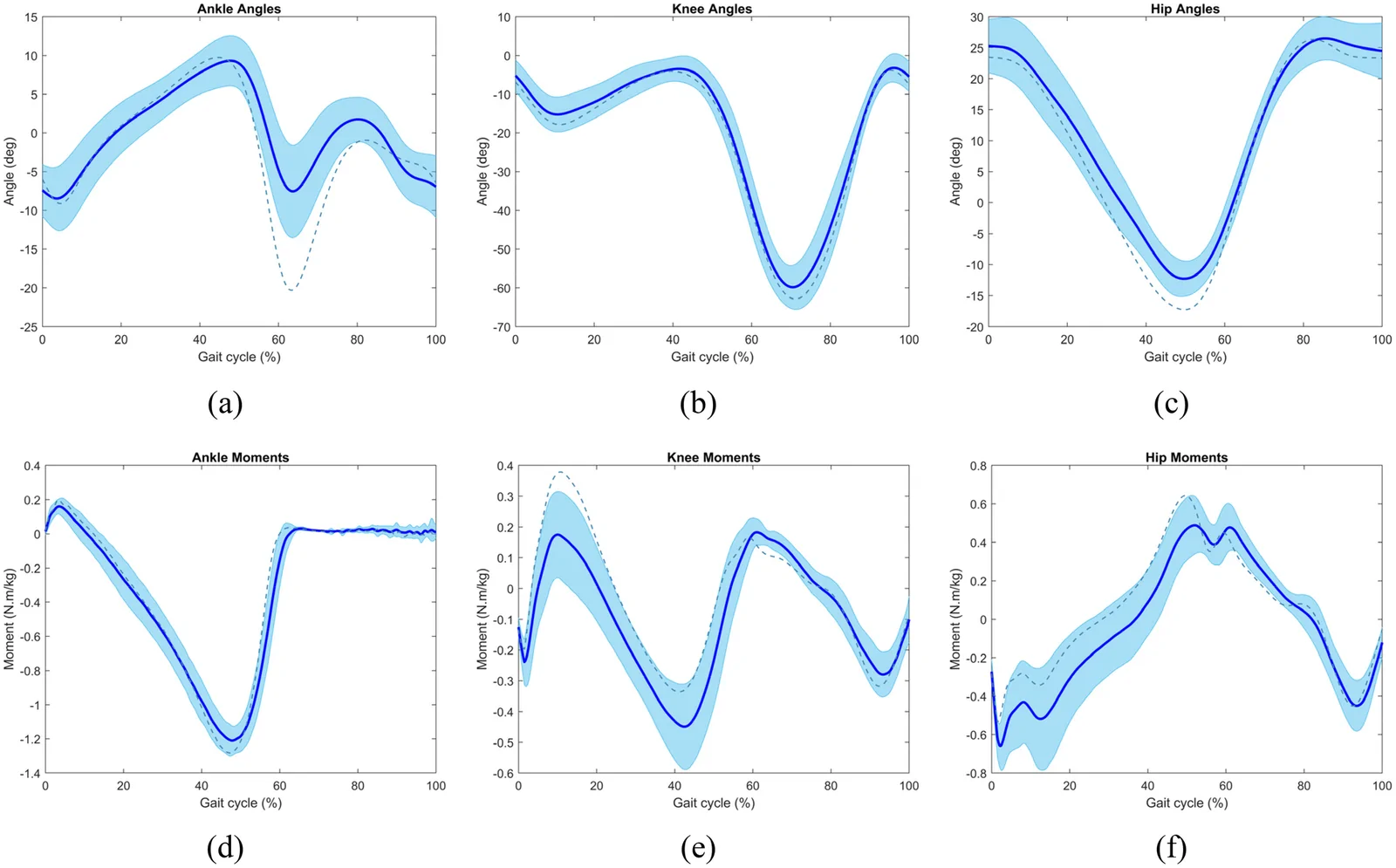
Joint kinematics (**a–c**) and kinetics (**d–f**)for the old group normalised to gait cycle – ensemble averages for the ankle (**a, d**), knee (**b, e**), and hip (**c, f**) joints (solid lines), along with the standard deviation (blue shading around the curves). The dotted lines represent the corresponding curves for the young group..

From Table 3, the older group has a 33.07% reduction in the ankle ROM (*F* = 16.34, *p* = 0.0008, 95% CI = [−16.99, −5.37], *g* = −1.73) and a 9.02% reduction in the hip joint ROM (*F* = 4.48, *p* = 0.0486, 95% CI = [−8.30, −0.03], *g* = −0.91). Peak ankle plantarflexion was found to decrease with age by 47.87% (*F* = 12.72, *p* = 0.0022, 95% CI = [4.52, 17.47], *g* = 1.53), along with the peak hip extension which decreased by 24.72% (*F* = 10.04, *p* = 0.0053, 95% CI = [1.55, 7.63], *g* = 1.36) (Figure 3).

**Table 3:** Average ± standard deviation of the kinematic parameters, in which a statistically significant difference was obtained, for both age groups.

Kinematic parameter (deg)	Young group	Older group
Ankle ROM	33.81 ± 8.06	22.63 ± 3.40
Hip ROM	46.15 ± 4.28	41.99 ± 4.52
Peak ankle plantarflexion	−22.96 ± 8.60	−11.97 ± 4.59
Peak hip extension	−18.56 ± 3.46	−13.98 ± 3.00

#### 3.1.3 Kinetic parameters

The peak knee moment was found to be on average 31.69% lower (0.27 ± 0.14 vs. 0.39 ± 0.06 Nm/kg) for the older group compared to the younger one (*F* = 6.53, *p* = 0.0198, 95% CI = [−0.23, −0.02], *g* = −1.09), and more likely to be delayed (*F* = 23.66, *p* = 0.0001, 95% CI = [15.73, 39.65], *g* = 2.08) from around 10.85%–60.63% stride. Peak hip moment is very likely to be delayed by an average of 13.79% (*F* = 16.89, *p* = 0.0007, 95% CI = [3.41, 10.53], *g* = 1.76) from around 50.55%–57.52% stride.

#### 3.1.4 Muscle activations

A general trend of higher activation levels was observed among all the considered lower limb muscle groups on older subjects, as shown in Table 4.

**Table 4:** Relative differences between age groups in average and integrated activations for each muscle group.

Muscle group	Average activations		Integrated activations	
	Relative difference	*p*-value	Relative difference	*p*-value
Hamstrings	+56.93%	0.0052	+52.44%	0.0101
Glutes	+62.06%	0.0001	+58.73%	0.0002
Hip adductors	+42.54%	0.006	+39.42%	0.0082
Hip flexors	+27.41%	0.008	+24.68%	0.0144
Hip extensors	+54.97%	0.0024	+51.35%	0.0043
Knee flexors	+63.85%	0.0003	+59.61%	0.0006
Knee extensors	+25.35%	0.0168	+23.36%	0.0424
Plantarflexors	+55.13%	3.02 × 10^−5^	+51.80%	5.67 × 10^−5^
Dorsiflexors	+39.02%	0.0003	+36.71%	0.003

### 3.2 Comparison of the ankle assistance techniques

#### 3.2.1 Bilateral assistance

Here the conditions are PF, DF, and PFDF, and the pairwise comparisons were done in order (PF vs. DF, PF vs. PFDF, and DF vs. PFDF). Significant differences were obtained for all parameters (*p* < 0.001). While the PF and DF devices provide similar savings in muscle activations musc% (*p* = 1, 95% CI = [−1.85, 1.46], Cohen's dz = −0.09), the PFDF device provides nearly 50% more savings (*p* < 0.001, 95% CI = [1.96, 3.62] and [1.90, 4.07], Cohen's dz = 2.40 and 1.97 for PF–PFDF and DF–PFDF). For metabolic savings, a significant difference is only present between DF and PFDF which provides the highest savings (*p* < 0.001, 95% CI = [1.79, 2.68], Cohen's dz = 3.63 for ga_metab_reduc and *p* = 0.0053, 95% CI = [−0.21, −0.07], Cohen's dz = −1.38 for abs_ga_metab_reduc). The COT values for PF (*p* < 0.001, 95% CI = [0.13, 0.21], Cohen's dz = 2.75) and PFDF (*p* = 0.0015, 95% CI = [0.14, 0.32], Cohen's dz = 1.85) are significantly lower than those obtained in the unassisted scenario, and the PFDF value is lower than the DF one (*p* < 0.001, 95% CI = [0.11, 0.19], Cohen's dz = 2.97). PFDF has the highest peak positive power (vs. PF: *p* = 0.0286, 95% CI = [−0.81, −0.15], Cohen's dz = −1.04; vs. DF: *p* = 0.0176, 95% CI = [−1.42, −0.32], Cohen's dz = −1.13). PF and PFDF have similar average positive power (*p* = 1, 95% CI = [−0.19, 0.27], Cohen's dz = 0.13), while DF has a significantly lower value (*p* < 0.001, 95% CI = [−0.34, −0.15], Cohen's dz = −1.81). In terms of efficiency, PF achieves a similar value to PFDF (*p* = 1, 95% CI = [−0.03, 0.02], Cohen's dz = −0.17), while for performance, PF and DF have similar results (*p* = 1, 95% CI = [−0.06, 0.10], Cohen's dz = 0.19).

#### 3.2.2 Unilateral assistance

In this case, the conditions are PFr, DFr, PFDFr, PFl, DFl, and PFDFl, and the pairwise comparisons were done in order. Significant differences were obtained for all parameters (*p* ≤ 0.0011). No significant differences were found for PFr-PFl (except for a 2% difference in COT, *p* = 0.0462, 95% CI = [0.06, 0.20], Cohen's dz = 1.34), DFr-DFl, and PFDFr-PFDFl (*p* = 1). In general, PFDF provides more musc% savings than DF (DFr-PFDFr: *p* = 0.0103, 95% CI = [0.93, 2.43], Cohen's dz = 1.60, DFl-PFDFl: *p* = 0.0097, 95% CI = [0.78, 2.03], Cohen's dz = 1.61) or PF (PFl-PFDFr *p* = 0.0031, 95% CI = [−2.47, −1.11], Cohen's dz = −1.89, PFl-PFDFl *p* = 0.0053, 95% CI = [0.99, 2.35], Cohen's dz = 1.76) (see Supplementary Table S2 in the supplementary material for full results).

#### 3.2.3 Unilateral vs. bilateral

##### 3.2.3.1 Plantarflexion assistance

Here the conditions are PFr, PFl, and PF, and the pairwise comparisons were done in order (PFr vs. PFl, PFr vs. PF, and PFl vs. PF). All parameters exhibited significant differences (*p* ≤ 0.0012). The bilateral exoskeleton provides the most musc% savings (compared to PFr: *p* = 0.0027, 95% CI = [0.79, 2.17], Cohen's dz = 1.53 and PFl: *p* < 0.001, 95% CI = [1.24, 2.85], Cohen's dz = 1.82), ga_metab_reduc (compared to PFr: *p* < 0.001, 95% CI = [0.58, 1.36], Cohen's dz = 1.77 and PFl: *p* = 0.0022, 95% CI = [0.87, 2.31], Cohen's dz = 1.58), abs_ga_metab_reduc (compared to PFr: *p* = 0.0011, 95% CI = [−0.10, −0.04], Cohen's dz = −1.75 and PFl: *p* = 0.0027, 95% CI = [−0.17, −0.06], Cohen's dz = −1.54), and COT (compared to PFr: *p* < 0.001, 95% CI = [0.04, 0.09], Cohen's dz = 1.92 and PFl: *p* = 0.0023, 95% CI = [0.06, 0.16], Cohen's dz = 1.57); however, it has a higher peak positive power (*p* = 0.0015, 95% CI = [−1.55, −0.62], Cohen's dz = −1.67), and average positive power (*p* = 0.040, 95% CI = [−0.65, −0.10], Cohen's dz = −0.97) relative to the left-only device, but similar average positive power to the right device (*p* = 1, 95% CI = [−0.35, 0.23], Cohen's dz = −0.15). The bilateral device is significantly more efficient (*p* < 0.001, 95% CI = [−0.03, −0.01], Cohen's dz = −2.12) and better performing (*p* < 0.001, 95% CI = [−0.11, −0.07], Cohen's dz = −3.15) than the right unilateral device, but has a similar performance to the left-only device (*p* = 1, 95% CI = [−0.13, 0.13], Cohen's dz = 0.00).

##### 3.2.3.2 Dorsiflexion assistance

The assessed conditions are DFr, DFl, and DF, and the pairwise comparisons were done in order (DFr vs. DFl, DFr vs. DF, and DFl vs. DF). All parameters had a significant difference (*p* < 0.001) except for ga_metab_reduc. The unilateral devices provided similar musc% savings (*p* = 1, 95% CI = [−0.91, 1.21], Cohen's dz = 0.10) while the bilateral one provided the most savings (compared to DFr: *p* < 0.001, 95% CI = [1.04, 2.44], Cohen's dz = 1.77 and DFl: *p* = 0.0016, 95% CI = [0.90, 2.27], Cohen's dz = 1.66). The two unilateral devices provided similar (*p* = 1) ga_metab_reduc (95% CI = [−0.90, 0.74], Cohen's dz = −0.07), abs_ga_metab_reduc (95% CI = [−0.03, 0.04], Cohen's dz = 0.07), and COT (95% CI = [−0.06, 0.05], Cohen's dz = −0.05), with the bilateral actuators resulting in a significantly higher abs_ga_metab_reduc (*p* = 0.0087, 95% CI = [−0.08, −0.02], Cohen's dz = −1.28). The bilateral device has a higher peak positive power than the left-only device (*p* = 0.0157, 95% CI = [−1.25, −0.29], Cohen's dz = −1.16). A higher average positive power was required for the bilateral device compared to its unilateral counterparts (compared to DFr: *p* = 0.0170, 95% CI = [−0.30, −0.07], Cohen's dz = −1.14 and DFl: *p* < 0.001, 95% CI = [−0.31, −0.17], Cohen's dz = −2.55). A similar efficiency was obtained for both left-only and bilateral devices (*p* = 1, 95% CI = [−0.04, 0.05], Cohen's dz = 0.14). The bilateral device had a similar performance (*p* = 1) to both unilateral devices (compared to DFr: 95% CI = [−0.09, 0.06], Cohen's dz = −0.15 and DFl: 95% CI = [−0.13, 0.22], Cohen's dz = 0.22).

##### 3.2.3.3 Plantarflexion and dorsiflexion assistance

Here the conditions are PFDFr, PFDFl, and PFDF, and the pairwise comparisons were done in order (PFDFr vs. PFDFl, PFDFr vs. PFDF, and PFDFl vs. PFDF). Significant differences were obtained for all parameters (*p* < 0.001). While the unilateral devices resulted in similar (*p* = 1) musc% (95% CI = [−1.27, 1.02], Cohen's dz = −0.08), ga_metab_reduc (95% CI = [−1.51, 0.90], Cohen's dz = −0.18), abs_ga_metab_reduc (95% CI = [−0.05, 0.10], Cohen's dz = 0.25), and COT (95% CI = [−0.10, 0.05], Cohen's dz = −0.24), the bilateral exoskeleton had better savings than both for all four variables: musc% (compared to PFDFr: *p* < 0.001, 95% CI = [2.23, 3.85], Cohen's dz = 2.68 and PFDFl: *p* < 0.001, 95% CI = [2.42, 3.92], Cohen's dz = 3.02), ga_metab_reduc (compared to PFDFr: *p* = 0.0047, 95% CI = [0.77, 2.36], Cohen's dz = 1.41 and PFDFl: *p* = 0.0029, 95% CI = [0.99, 2.75], Cohen's dz = 1.52), abs_ga_metab_reduc (compared to PFDFr: *p* = 0.0042, 95% CI = [−0.17, −0.06], Cohen's dz = −1.44 and PFDFl: *p* = 0.0048, 95% CI = [−0.21, −0.07], Cohen's dz = −1.41), and COT (compared to PFDFr: *p* = 0.0030, 95% CI = [0.05, 0.15], Cohen's dz = 1.51 and PFDFl: *p* = 0.0041, 95% CI = [0.06, 0.19], Cohen's dz = 1.44). The bilateral device has a higher peak positive power than the right-only (*p* = 0.0235, 95% CI = [−0.93, −0.19], Cohen's dz = −1.08) and left-only (*p* = 0.0057, 95% CI = [−1.63, −0.51], Cohen's dz = −1.37) devices, and a higher average positive power than both unilateral devices (compared to PFDFr: *p* = 0.0090, 95% CI = [−0.38, −0.11], Cohen's dz = −1.27, and PFDFl: *p* < 0.001, 95% CI = [−0.52, −0.29], Cohen's dz = −2.52). The unilateral exoskeletons have similar efficiency (*p* = 1, 95% CI = [−0.02, 0.03], Cohen's dz = 0.07), whereas the bilateral device has a higher efficiency than the left-only actuators (*p* = 0.0054, 95% CI = [−0.04, −0.01], Cohen's dz = −1.53). The unilateral devices have similar performance (*p* = 1, 95% CI = [−0.13, 0.11], Cohen's dz = −0.08), as well as the bilateral and left-only devices (*p* = 1, 95% CI = [−0.03, 0.02], Cohen's dz = −0.09).

## 4 Discussion

### 4.1 Key assistance parameters

There has been a recent increase of interest in providing assistance to older adults and helping them carry on their daily activities, stay active, and live independently with a good quality of life, which also alleviates the burden on the healthcare system. Thus, efforts are being targeted towards the development of assistive technologies such as exoskeletons, as traditional assistive devices fail to provide high-level assistance in a practical form factor (30).

Exoskeletons for mobility assistance in older adults come under the non-medical/augmentation category, as healthy older adults have reduced physical abilities but do not actually have a motor disease that requires medical assistance or body-weight support (30). The ultimate goal of any intervention is to promote locomotor features typical of normal gait (87), hence the relevance of this study in comparing young and elderly gait and extracting key assistance parameters.

Lower limb exoskeletons target one or more of the hip, knee, and ankle joints. Exoskeletons adopt different control strategies, and often focus on either position or force/torque control (33). In position control, the device drives the limb following a trajectory of desired joint angles based on data from healthy individuals (41). Other devices focus on augmenting the biological torque of a joint by providing torque assistance, often using predefined timed profiles (33, 88, 89). In the context of the present study, we suggest that position assistance might be beneficial for joints with limited ROMs, while torque assistance could help in the case of joints with reduced peak moments. Hence, in the case of healthy old adults, position assistance is needed at the ankle, enhancing the range of motion, in particular plantarflexion. In fact, many ankle exoskeletons assist both degrees of freedom at the ankle (plantar- and dorsiflexion) (59, 90), and many provide plantarflexion assistance only (49, 56). On the other hand, torque assistance is required at the knee, possibly using timed assistance profiles to provide assistive torques and restore the peak that is observed among the younger population in early stance. At the hip level, position assistance to enhance the ROM (extension in particular) is needed alongside torque support (timed at around 50% stride). Exoskeletons that are able to provide assistive torques at the knee (91, 92) and the hip (93) have previously been developed.

Additionally, the observed spatiotemporal changes, in terms of spending a longer proportion of the stride in stance and double support, and having shorter steps and stride distance, suggest the need for balance and postural control support, which can also be provided by exoskeletons (34, 35).

Furthermore, the simulated actuators were implemented as ideal path actuators with a high optimal force in the CMC optimization, encouraging the solver to preferentially use the device rather than the muscles whenever mechanically advantageous. As a result, the predicted absolute reductions in muscle effort and metabolic cost may represent an upper bound compared with a real exoskeleton having finite actuator force, power, bandwidth, and control limitations. However, because identical actuator assumptions and optimization settings were used for all simulated assistance strategies, the comparative evaluation between plantarflexion, dorsiflexion, and combined assistance is expected to remain valid.

Even though the old-young comparison did not show a specific pattern in muscle activation increase, there was a generally increasing trend; previously-published results include (94), which considered fewer muscles (vastus lateralis, biceps femoris, tibialis anterior, and gastrocnemius lateralis), and compared averaged EMG linear envelopes to find a 67.3% increase in muscle activations in older subjects. Reducing muscle activations can also be achieved with exoskeleton assistance (36).

However, human walking is a complex movement, as joints are interrelated and tend to compensate for each other (10), and there are several important considerations when it comes to what parameters to target and optimize during exoskeleton design, such as metabolic cost, which has become a standard for evaluating the benefits provided exoskeletons (45, 95). Therefore, implementing the suggestions we gave in this paper could be beneficial to restore young-age gait, but they might either affect each other (i.e., imposing a desired trajectory at one joint might cause another joint to compensate, making its kinematics/kinetics even worse) or other variables of interest such as metabolic consumption, or comfort and compliance.

Other limitations of this study relate to the choice of the target population, which consisted of healthy, able-bodied older adults, explicitly free of motor disease. While this choice allows for isolating age-related biomechanical changes, the ultimate clinical target population for lower-limb exoskeletons often includes frail older adults who are frailer and suffer from comorbidities, such as osteoarthritis, sarcopenia, and reduced skin/vascular integrity. Therefore, the benefits reported in this study as well as the design guidelines may need to be reviewed separately in less healthy cohorts before broader clinical translation. Moreover, as aging makes the skin thinner and less tolerant of concentrated mechanical loading at the device interface compared to younger adults, with actual (non-massless) bilateral ankle devices adding distal mass, there is a chance to increase inertial demand on the hip flexors and potentially inducing proximal fatigue with prolonged use; however, we believe that careful design choices placing the heavy parts of the device nearer the body center of mass should mitigate this concern (51, 96). And while we attempted to have two very distant age groups for the young-old comparison, the old group still spans 14 years (72–86 years), encompassing meaningful physiological heterogeneity between “young-old” and “old-old” individuals, who can differ in muscle strength, balance capacity, and fall risk even within a healthy, able-bodied population; therefore, it might be worth considering a smaller age range for more refined comparisons (e.g., 72–79 vs. 80–86 years) in future work.

Furthermore, it is worth noting that, while COT is widely used in scientific literature as an indicator of the energy required for a movement, correlating with exertion and fatigue, its validity as a clinical measure of the perceived exertion is inevitably affected by the influence of the specific exoskeleton, which adds sensory weight and requires motor adaptation. However, further analysis of this limitation falls outside the scope of the present work. Hence, this study serves as a guideline for future work, but experimental evaluation of exoskeletons is a must to ensure the efficacy of a chosen design.

### 4.2 Evaluating different ankle assistance techniques

For easier interpretability of the above results, where a significant difference is present, we assign the value 1 to the better device category and −1 to the worse; in case of significant similarity (*p* > 0.95), the value 0 is assigned. Then, realizing that the three parameters ga_metab_reduc, abs_ga_metab_reduc, and COT all report on metabolic expenditure, the previously obtained values are averaged over the 3 parameters. Therefore, the comparison between devices now consists of the sum of 6 factors, which have been frequently used in the literature to assess the performance of ankle exoskeletons: (1) muscle activation savings, (2) metabolic expenditure savings, (3) device weight (simulated by peak positive power), (4) device power requirements (approximated by average positive power), (5) efficiency, and (6) performance. In fact (97–99), used muscle activity, and metabolic consumption is one of the most common assessment methods based on indirect calorimetry data, as in (45, 48, 95, 97, 100, 101). Device weight is an important factor to be optimised in exoskeleton design, as increasing weight affects gait [increased step length, decreased step height (102)] and balance (103) and increases metabolic consumption, especially when the weight is added distally on the legs (33, 51, 96). Power requirements affect battery life, which is crucial in wearable exoskeletons, and can range from 30 min (45) to 5 h (104). Moreover, performance is inspired by (86)'s muscle-tendon efficiency and (105)'s performance index (61).

#### 4.2.1 Bilateral assistance

Looking at Table 5, all the bilateral exoskeletons have mostly similar efficiency and performance. The PFDF device is the most beneficial in terms of muscle activations and metabolic savings, however, it lags on power requirements and device weight. Therefore, the DF and PFDF can be given the same rating, as DF provides less muscular and metabolic improvements but with lower power demands.

**Table 5:** Evaluation of the bilateral assistance devices.

Factor	PF	DF	PFDF
Muscle activations	−1	−1	2
Metabolic savings		−1	1
Device weight	1	1	−2
Power requirements	−1	1	−1
Efficiency	0		0
Performance	0	0	
Total	−1	0	0

**Figure 4:**
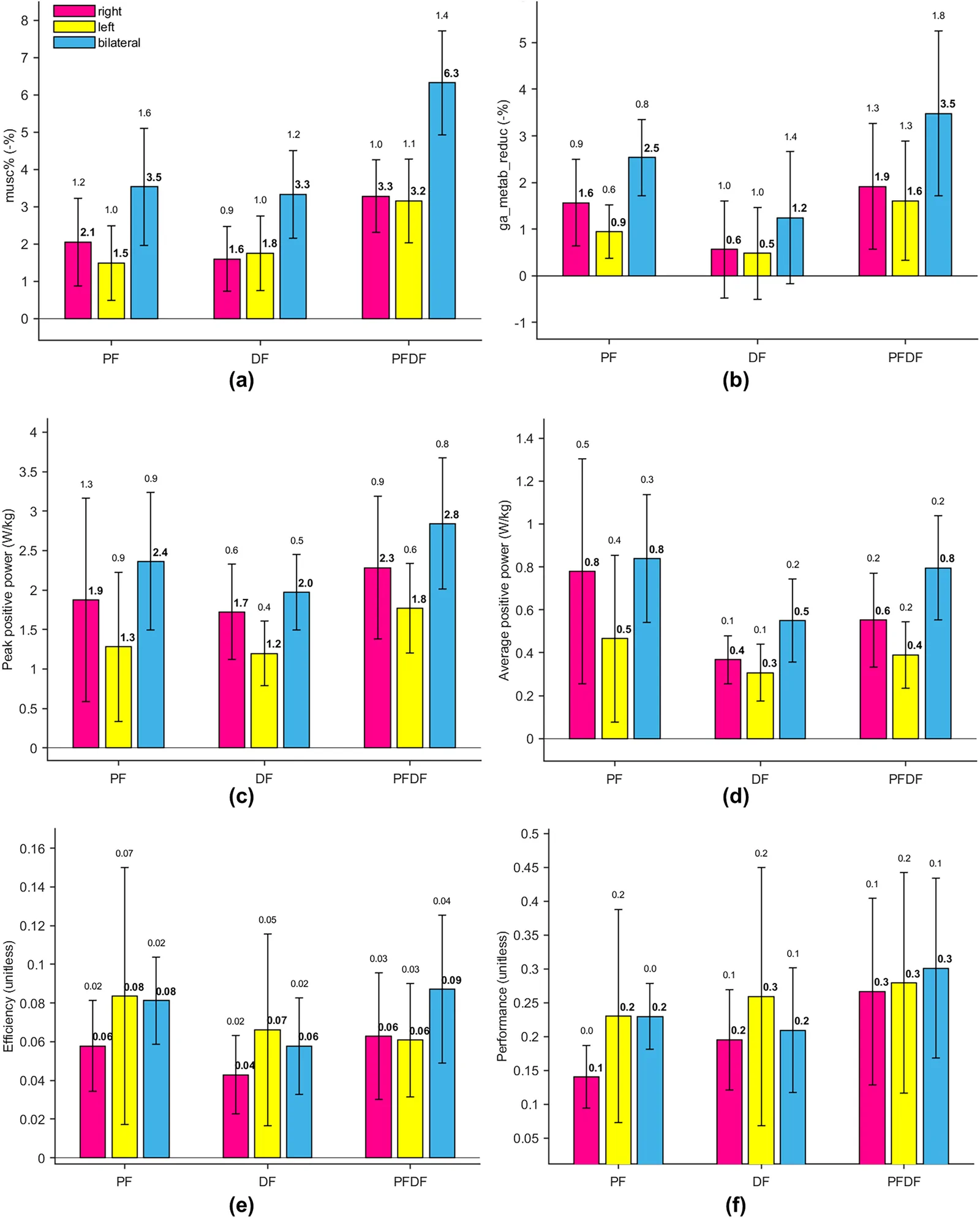
Average and standard deviation of the **(a)** musc%, **(b)** ga_metab_reduc, **(c)** peak positive power, **(d)** average positive power, **(e)** efficiency, and **(f)** performance of the unilateral (right, left) and bilateral plantarflexion, dorsiflexion, and plantarflexion/dorsiflexion assistance devices across subjects.

However, looking at Figure 4b, DF can sometimes increase the metabolic consumption, hence PFDF is deemed best overall.

#### 4.2.2 Unilateral assistance

Referring to Table 6 and Supplementary Table S2F, no significant differences are observed between unilateral devices achieving the same function—PFr-PFl, DFr-DFl, and PFDFr-PFDFl—which validates our study by balancing between both sides (no dominant side in the experiments). It is also interesting to see that there are no significant differences between PFr-DFr, PFr-DFl, DFr-PFl, and PFl-DFl, which suggests that unilateral plantarflexion or dorsiflexion assistance can be equally beneficial. Differences are observed in the cases where a plantarflexion/dorsiflexion device is compared with a plantarflexion-only or dorsiflexion-only device, with the former having an advantage in terms of muscle activations and/or metabolic cost, correlating with the bilateral assistance results in Section 4.2.1 above.

**Table 6:** Evaluation of the unilateral assistance devices.

Factor	PFr	PFl	DFr	DFl	PFDFr	PFDFl
Muscle activations	−1	−2	−1	−1	3	2
Metabolic savings			−1	−1	1	1
Device weight						
Power requirements						
Efficiency	0	0	0	0	0	0
Performance	0	0	0	0	0	0
Total	−1	−2	−2	−2	4	3
Average	−1.5		−2		3.5	

Similar to the bilateral case, unilateral PFDF assistance provides the most muscular and metabolic enhancement, this time without a significant additional power and weight burden compared to the other unilateral actuators, making it the best overall. All unilateral devices seem to have similar efficiency and performance. Averaging the right and left sides for each assistance direction, PFDF appears to be the best unilateral exoskeleton, followed by PF and DF.

#### 4.2.3 Unilateral vs. bilateral

##### 4.2.3.1 Plantarflexion assistance

As shown in Table 7, bilateral PF assistance is more beneficial in terms of muscle activations and metabolic consumption and predicted efficiency and performance, despite having higher power requirements and associated device weight, eventually making bilateral devices better than unilateral ones for plantarflexion assistance.

**Table 7:** Evaluation of the plantarflexion assistance devices.

Factor	PFr	PFl	PF
Muscle activations	−1	−1	2
Metabolic savings	−1	−1	2
Device weight		1	−1
Power requirements	−1	1	−1
Efficiency	−1		1
Performance	−1	1	1
Total	−5	1	4
Average	−2		4

##### 4.2.3.2 Dorsiflexion assistance

Dorsiflexion assistance devices have similar efficiency and performance regardless of being unilateral or bilateral. Bilateral DF, despite providing the best muscle and metabolic enhancements, lags on power requirements and device weight. Eventually, even though Table 8 gives a slight advantage to unilateral devices, the results can be seen as inconclusive, as DFr appears better than DF while DFl appears worse, which could be attributed to some minor subject-specific details such as the choice of leading foot.

**Table 8:** Evaluation of the dorsiflexion assistive devices.

Factor	DFr	DFl	DF
Muscle activations	−1	−1	2
Metabolic savings	−0.3333	−0.3333	1
Device weight		1	−1
Power requirements	1	1	−2
Efficiency		0	0
Performance	0	0	0
Total	−0.3333	0.6667	0
Average	0.1667		0

##### 4.2.3.3 Plantarflexion and dorsiflexion assistance

When assisting both plantarflexion and dorsiflexion, the bilateral PFDF exoskeleton is more beneficial in terms of muscle activations and metabolic expenditure savings and has the best efficiency (Table 9). PFDF is expected to be heavier and have higher power requirements, but performance is expected to be similar across all devices than its unilateral counterparts. Overall, bilateral PFDF emerges as the best device.

**Table 9:** Evaluation of the devices providing both plantarflexion and dorsiflexion assistance.

Factor	PFDFr	PFDFl	PFDF
Muscle activations	−1	−1	2
Metabolic savings	−1	−1	2
Device weight	1	1	−2
Power requirements	1	1	−2
Efficiency	−1	−1	1
Performance	0	0	0
Total	−1	−1	1
Average	−1		1

#### 4.2.4 Summary

Table 10 summarises the results of the present work. Even if dorsiflexion-only assistance seems appealing in terms of device weight and power demands, it might lead to an increased metabolic consumption (Figure 4b). This can be associated with the fact that ankle gait kinematics in older adults show a decreased plantarflexion but no significant difference in dorsiflexion compared to younger adults (Section 3.1.2, Table 3).

**Table 10:** Summary of the results.

Factor	Bilateral	Unilateral	Unilateral vs. bilateral		
			Plantarflexion	Dorsiflexion	Both
Best savings	PFDF	PFDF	Bilateral	Bilateral	Bilateral
Best Power/weight	DF	PF/DF	Unilateral	Unilateral	Unilateral
Best overall	PFDF	PFDF	Bilateral	–	Bilateral

Providing both plantarflexion and dorsiflexion assistance is best, whether uni- or bilateral. Bilateral assistance is also best, regardless of the assisted direction (plantarflexion, dorsiflexion, or both). That said, it is worth keeping in mind that PFDF and bilateral devices can have higher power requirements and weight. This correlates with (33)'s complexity index for ankle exoskeletons, in particular the fact that device weight tends to increase with increasing complexity, with the number of assisted angles and limbs both contributing to an increasing complexity. Nevertheless, there are examples of bilateral PFDF devices weighing around 2 kg (48, 100), which is quite light compared to a 10 kg bilateral device (106) for reference [heaviest reported in (33)], which makes it a matter of design optimisation, and a motive to shift towards soft exoskeletons based on lightweight materials, similar to (48, 100).

In this study we used massless lossless actuators with unlimited torque instead of a complete exoskeleton model with weight, and assumed fixed kinematics and ground reaction from the unassisted data to simulate the assistance; however, we managed to simulate the real actuators as closely as possible by using unidirectional path actuators instead of coordinate actuators, and future work shall look into better ways to simulate exoskeleton assistance, such as predictive simulations (107).

Furthermore, it is worth noting that benefits from exoskeleton assistance should not be expected from the first device use, as an initial adaptation period is often required, especially for older adults who often present with reduced proprioceptive acuity and slower motor learning relative to younger adults, in order to ensure optimal benefits (29). Nonetheless, user adherence and device acceptance is an important factor to be considered when developing exoskeleton prototypes, to ensure that the target user actually ends up using the device as much as intended.

Nevertheless, the study is intended for an initial screening of the different assistance configurations, and the original objective was reached: the hypothesis on bilateral assistance being superior to its unilateral counterpart was verified, and we managed to identify that assisting both angles, i.e., plantarflexion and dorsiflexion, is the most beneficial for older adults.

## 5 Conclusion

This study highlights key biomechanical changes associated with aging gait and translated these findings into design considerations for targeted lower-limb exoskeleton assistance. Gait analysis of older adults demonstrated reduced ankle and hip range of motion, decreased plantarflexion, delayed joint kinetics, altered spatiotemporal gait parameters, and increased muscle activation demands, all of which are associated with reduced walking efficiency and increased fall risk. Therefore, we suggest that position control at the ankle and hips could help enhance the range of motion, while torque assistance with timed profiles at the knees and hips can contribute to restoring normal gait patterns. Additionally, balance support plays a critical role in the reduction of fall and injury risks in older adults. Based on musculoskeletal simulations, we evaluated multiple ankle exoskeleton configurations representative of cable-driven wearable systems, using six factors that represent muscle activation, metabolic consumption, device weight and power requirement, efficiency, and performance. This way we demonstrated that combined plantarflexion and dorsiflexion assistance produces the greatest reductions in muscle activation and metabolic expenditure, regardless of whether it is done unilaterally or bilaterally, while bilateral assistance consistently outperforms the unilateral configurations. Although dorsiflexion-only assistance reduces device power requirements, it could also increase metabolic cost in some subjects, highlighting the importance of balancing assistance benefits against energetic efficiency. The findings of this study provide a framework and actionable guidelines for the design and development of lightweight and energy-efficient assistive exoskeletons aimed at enhancing mobility, independence, and healthy aging while reducing fall-related injuries in older adults.

## Data Availability

The raw data supporting the conclusions of this article will be made available by the authors, without undue reservation.
