# Supplementary material for: Lower limb exoskeletons for older adults: key assistance parameters and evaluation of ankle assistive strategies via musculoskeletal modelling
Source: Front Sports Act Living. 2026 Aug 28;8:1888675. doi: 10.3389/fspor.2026.1888675 (PMC13563653; doi:10.3389/fspor.2026.1888675)
Supplement: Supplementary file 1 [file Datasheet1.pdf]

Supplementary Material

1. Algorithm for EMG-Constrained CMC

The CMC tool in OpenSim makes use of a constraints file, which can be used to set default values as well as limits to the actuator excitations using minimum and maximum nodes between which the estimated muscle activation is allowed to vary.

In the first instance (v1), rough manual adjustment of maximum nodes was performed, while fixing minimum nodes at 0.02, as the mathematical muscle models used in OpenSim are often not as well behaved for near zero excitation, and CMC tool was run; rough manual adjustment was then performed for minimum nodes in a second instance (v2) to envelope the signal more closely. Then, instead of manually adjusting the constraints, which can be tedious and not very exact, a MATLAB script was written to convert each muscle's EMG data into sets of minimum and maximum constraints (v3). The algorithm envelopes the EMG signal between max and min lines by finding the maximum and minimum EMG amplitude in windows of 5 ms. Then, nodes are assigned to the start and end of each of the 5 ms intervals. To minimize the number of constraints, for series of consecutives nodes that are within 1 mV of each other, the middle nodes are discarded as long as the envelopes are still wrapping the EMG signal. The minimum nodes were limited to 0.01 minimum. Again, when 3 or more consecutive values are limited to 0.01, the middle nodes are removed to minimise the number of nodes/constraints. The script outputs 2 files: min_nodes.txt and max_nodes.txt, which contents are placed in the corresponding sections in the CMC constraints file.

For one subject (SUBJ01), all 3 constraint versions (v0, v1, v2, v3) were simulated using the unmodified Gait2392 model. The obtained excitations were smoothened by a moving average filter (n = 51) and compared against the actual EMG. Muscle parameters were then changed to reflect the impacts of ageing as described in 2.5, and the CMC tool was executed (v_old). Eventually, v3 and v_old results were compared to adjusted EMG data, limited to a minimum of 0.01 to match the constraints (v3 adj and v_old adj). Only v0, v3, v3 adj, v_old and v_old adj were tested in 4 subjects (SUBJ02, SUBJ05, SUBJ08, and SUBJ22).

To evaluate the match between the computed excitations and the actual EMG data, the relative error (RE), mean absolute error (MAE), mean squared error (MSE), normalized mean absolute error (NMAE), and root mean squared error (RMSE) were computed.

1.1. Results

Visually inspecting the muscle excitations for SUBJ01's right tibialis anterior muscle (Figure): the v0 estimations are very different from the actual EMG. The v1 and v2 estimations got closer to the EMG line, and v3 is an even better match. Table 3 shows the average error reduction compared to v0 over the 5 subjects, proving that our algorithm results in a significant reduction of the error and hence a smaller difference between the computed excitations and the actual EMG.

There is no significant difference in the errors obtained when using the unmodified model or the age-adjusted model (with v_old having a slight advantage).

	v3	v3 adj	v_old	v_old adj
**RE**	95.04	96.56	95.09	96.61
**MAE**	91.88	92.28	91.95	92.35
**NMAE**	79.00	81.17	79.05	81.23
**MSE**	99.10	99.11	99.13	99.14
**RMSE**	91.35	91.47	91.48	91.60

Table S1: Relative error reduction (percentage %) between EMG-constrained CMC (versions v3, v3 adj, v_old, and v_old adj) and default constraints (v0).

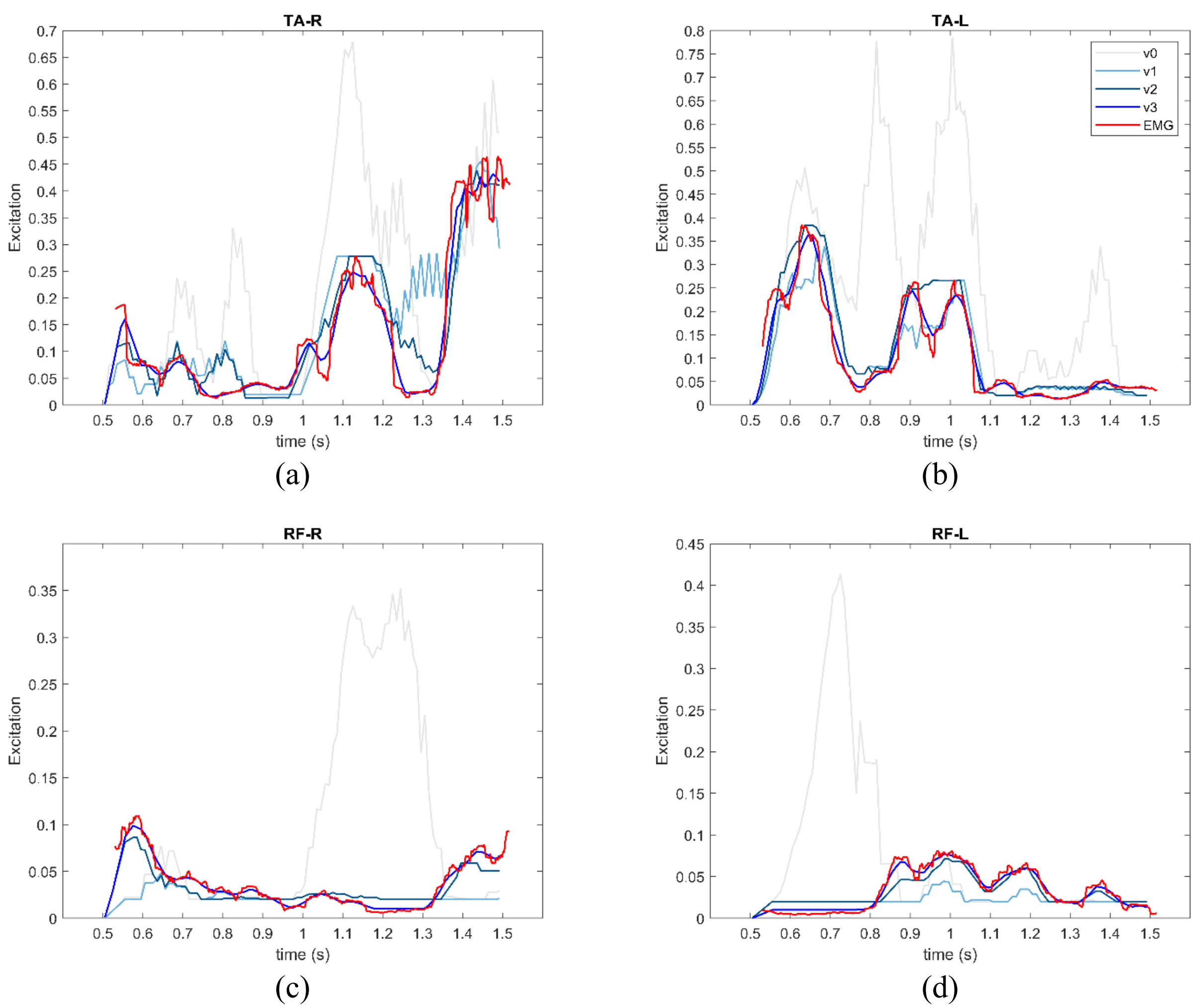

Figure S1: Muscle excitations computed by the CMC tool in OpenSim in versions v0, v1, v2, and v3 for SUBJ01's right ((a) TA-R) and left ((b) TA-L) tibialis anterior and right ((c) RF-R) and left ((d) RF-L) rectus femoris muscles. The actual processed EMG signal is plotted in red.

2. Unilateral Assistance Pairwise Comparison Results

Table S2: Results of the pairwise comparisons among the unilateral devices – the parameters, paired conditions and relative difference between them, the 95% confidence interval [CI95_Low, CI95_High], Cohen's dz and the Bonferroni-corrected p. The conditions are numbered in order (1–6): PFr, DFr, PFDFr, PFl, DFl, PFDFl.

Parameter	Cond1	Cond2	RelDiff Cond1-Cond2	CI95_Low	CI95_High	Cohen's_dz	p_bonferroni
musc_percent	1	2	28.53593	-1.67319	0.76143	-0.2679	1
musc_percent	1	3	-37.3731	0.629456	1.821354	1.470934	0.017993
musc_percent	1	4	37.98906	-1.60011	0.46947	-0.39081	1
musc_percent	1	5	17.32718	-1.6759	1.069385	-0.15804	1
musc_percent	1	6	-34.909	-0.24772	2.450279	0.583995	1
musc_percent	2	3	-51.2768	0.928715	2.433851	1.598151	0.010302
musc_percent	2	4	7.354465	-0.83846	0.619577	-0.10739	1
musc_percent	2	5	-8.72032	-0.90835	1.213593	0.102904	1
musc_percent	2	6	-49.3597	0.564081	2.550235	1.121689	0.093664
musc_percent	3	4	120.3353	-2.46814	-1.11332	-1.89104	0.003113
musc_percent	3	5	87.34321	-2.81615	-0.24117	-0.84936	0.37445
musc_percent	3	6	3.934592	-1.26835	1.020097	-0.0776	1
musc_percent	4	5	-14.9736	-0.81196	1.336092	0.174548	1
musc_percent	4	6	-52.8289	0.98803	2.345171	1.756949	0.005306
musc_percent	5	6	-44.5218	0.780959	2.028114	1.61126	0.00974
ga_metab_reduc	1	2	177.8672	-1.8534	-0.15069	-0.84197	0.389056
ga_metab_reduc	1	3	-18.2122	-0.28857	0.985731	0.391366	1

Parameter	Cond1	Cond2	RelDiff Cond1-Cond2	CI95_Low	CI95_High	Cohen's_dz	p_bonferroni
ga_metab_reduc	1	4	65.78917	-1.57064	0.32825	-0.46804	1
ga_metab_reduc	1	5	224.9409	-2.08448	-0.08283	-0.77456	0.55191
ga_metab_reduc	1	6	-2.65703	-1.2494	1.334853	0.023656	1
ga_metab_reduc	2	3	-70.5659	0.766481	1.934768	1.654009	0.008124
ga_metab_reduc	2	4	-40.3351	-0.30282	1.064521	0.398503	1
ga_metab_reduc	2	5	16.9411	-0.90321	0.739983	-0.07106	1
ga_metab_reduc	2	6	-64.9678	0.177095	1.912451	0.861363	0.351881
ga_metab_reduc	3	4	102.7065	-2.02192	0.082373	-0.65935	1
ga_metab_reduc	3	5	297.2976	-2.41488	-0.4496	-1.04266	0.139069
ga_metab_reduc	3	6	19.01895	-1.51047	0.898765	-0.18163	1
ga_metab_reduc	4	5	95.99649	-1.16396	0.239028	-0.47161	1
ga_metab_reduc	4	6	-41.2851	-0.02057	1.348417	0.693856	0.838045
ga_metab_reduc	5	6	-70.0429	0.528867	1.723908	1.34852	0.031463
abs_ga_metab_reduc	1	2	56.93676	-0.01676	0.099511	0.509075	1
abs_ga_metab_reduc	1	3	-23.1206	-0.07173	0.003142	-0.65532	1
abs_ga_metab_reduc	1	4	71.87625	-0.02092	0.1163	0.49722	1
abs_ga_metab_reduc	1	5	65.8462	0.0075	0.083054	0.857372	0.359228
abs_ga_metab_reduc	1	6	-6.421	-0.08491	0.069259	-0.07262	1
abs_ga_metab_reduc	2	3	-51.0125	-0.13889	-0.01244	-0.85614	0.361532
abs_ga_metab_reduc	2	4	9.519431	-0.02445	0.037085	0.146845	1

Parameter	Cond1	Cond2	RelDiff Cond1-Cond2	CI95_Low	CI95_High	Cohen's_dz	p_bonferroni
abs_ga_metab_reduc	2	5	5.677089	-0.03414	0.041944	0.073408	1
abs_ga_metab_reduc	2	6	-40.3715	-0.10432	0.005928	-0.63843	1
abs_ga_metab_reduc	3	4	123.5661	0.010553	0.153417	0.821036	0.433666
abs_ga_metab_reduc	3	5	115.7226	0.019309	0.139836	0.944563	0.229138
abs_ga_metab_reduc	3	6	21.72185	-0.04947	0.102412	0.249351	1
abs_ga_metab_reduc	4	5	-3.50837	-0.04642	0.041599	-0.03921	1
abs_ga_metab_reduc	4	6	-45.5544	-0.09115	-0.01987	-1.11429	0.097165
abs_ga_metab_reduc	5	6	-43.5748	-0.1083	0.002098	-0.68816	0.862997
COT	1	2	-1.06696	-0.12631	-0.01492	-0.90703	0.389109
COT	1	3	0.348605	-0.01957	0.06506	0.384572	1
COT	1	4	-0.69772	-0.1067	0.014684	-0.54228	1
COT	1	5	-1.11782	-0.13709	-0.01095	-0.83955	0.551569
COT	1	6	-0.0331	-0.08046	0.076119	-0.01981	1
COT	2	3	1.430834	0.050934	0.135796	1.574078	0.015999
COT	2	4	0.373221	-0.02125	0.070474	0.383851	1
COT	2	5	-0.0514	-0.05529	0.048483	-0.04693	1
COT	2	6	1.045012	0.015739	0.121161	0.928948	0.34758
COT	3	4	-1.04269	-0.14093	0.003417	-0.68149	1
COT	3	5	-1.46133	-0.16152	-0.03202	-1.06916	0.170405
COT	3	6	-0.38038	-0.10034	0.050507	-0.23631	1

Parameter	Cond1	Cond2	RelDiff Cond1-Cond2	CI95_Low	CI95_High	Cohen's_dz	p_bonferroni
COT	4	5	-0.42304	-0.07255	0.016524	-0.44995	1
COT	4	6	0.669294	-6.36E-05	0.087743	0.71432	1
COT	5	6	1.096977	0.038786	0.104921	1.554442	0.017423
peak_positive_power	1	2	8.961744	-0.81795	1.126827	0.113617	1
peak_positive_power	1	3	-17.7942	-0.97052	0.157597	-0.51549	1
peak_positive_power	1	4	46.48827	-0.57188	1.763707	0.365039	1
peak_positive_power	1	5	56.77612	-0.28023	1.640285	0.506599	1
peak_positive_power	1	6	6.106092	-0.96209	1.178208	0.072234	1
peak_positive_power	2	3	-24.5553	-1.16351	0.041707	-0.66585	0.967951
peak_positive_power	2	4	34.4401	-0.26773	1.150674	0.445304	1
peak_positive_power	2	5	43.8818	-0.07026	1.121437	0.631007	1
peak_positive_power	2	6	-2.62078	-0.56098	0.468222	-0.06447	1
peak_positive_power	3	4	78.19695	0.304896	1.699849	1.02807	0.149711
peak_positive_power	3	5	90.71169	0.330469	1.84251	1.028051	0.149725
peak_positive_power	3	6	29.07369	-0.11056	1.139601	0.588828	1
peak_positive_power	4	5	7.022981	-0.80744	0.975677	0.067493	1
peak_positive_power	4	6	-27.5668	-0.91913	-0.05658	-0.8092	0.461126
peak_positive_power	5	6	-32.32	-1.10894	-0.035	-0.76198	0.589124
average_positive_power	1	2	112.1549	0.016842	0.806729	0.745862	0.640463
average_positive_power	1	3	41.20757	-0.05155	0.506176	0.58312	1

Parameter	Cond1	Cond2	RelDiff Cond1-Cond2	CI95_Low	CI95_High	Cohen’s_dz	p_bonferroni
average_positive_power	1	4	67.30825	-0.22797	0.854707	0.414106	1
average_positive_power	1	5	154.0345	0.079543	0.865086	0.860229	0.353953
average_positive_power	1	6	100.1314	-0.03756	0.817014	0.652475	1
average_positive_power	2	3	-33.4413	-0.32491	-0.04403	-0.93964	0.235006
average_positive_power	2	4	-21.1386	-0.35202	0.155185	-0.27761	1
average_positive_power	2	5	19.74008	-0.04196	0.16302	0.42247	1
average_positive_power	2	6	-5.66732	-0.10981	0.065696	-0.17981	1
average_positive_power	3	4	18.48391	-0.23298	0.405094	0.192958	1
average_positive_power	3	5	79.90146	0.076519	0.413484	1.040246	0.140777
average_positive_power	3	6	41.72855	-0.01747	0.342298	0.645885	1
average_positive_power	4	5	51.83619	-0.15626	0.474147	0.360728	1
average_positive_power	4	6	19.61839	-0.17537	0.328091	0.216989	1
average_positive_power	5	6	-21.2188	-0.15606	-0.00911	-0.8041	0.473488
efficiency	1	2	34.54932	-0.01425	0.043929	0.392077	1
efficiency	1	3	-7.92946	-0.01983	0.00988	-0.25748	1
efficiency	1	4	-30.8482	-0.07883	0.027276	-0.37348	1
efficiency	1	5	-12.6089	-0.0427	0.026022	-0.18652	1
efficiency	1	6	-4.90311	-0.02805	0.022088	-0.09136	1
efficiency	2	3	-31.5712	-0.05002	0.010386	-0.50433	1
efficiency	2	4	-48.6049	-0.09448	0.013244	-0.57966	1

Parameter	Cond1	Cond2	RelDiff Cond1-Cond2	CI95_Low	CI95_High	Cohen's_dz	p_bonferroni
efficiency	2	5	-35.049	-0.06711	0.020755	-0.40551	1
efficiency	2	6	-29.3219	-0.04581	0.010172	-0.48932	1
efficiency	3	4	-24.8926	-0.08243	0.040828	-0.25944	1
efficiency	3	5	-5.08247	-0.03952	0.0328	-0.07144	1
efficiency	3	6	3.286992	-0.02123	0.025229	0.066087	1
efficiency	4	5	26.37577	-0.05998	0.094861	0.173159	1
efficiency	4	6	37.51911	-0.03728	0.082875	0.291706	1
efficiency	5	6	8.817619	-0.03938	0.050099	0.092054	1
performance	1	2	-28.1935	-0.12898	0.018825	-0.57287	1
performance	1	3	-47.3393	-0.20819	-0.04402	-1.18089	0.113854
performance	1	4	-39.0322	-0.21761	0.037988	-0.54018	1
performance	1	5	-45.828	-0.26111	0.023767	-0.64041	1
performance	1	6	-49.704	-0.26143	-0.01583	-0.86778	0.471808
performance	2	3	-26.6631	-0.18219	0.040136	-0.49114	1
performance	2	4	-15.0944	-0.18253	0.113069	-0.18063	1
performance	2	5	-24.5585	-0.1969	0.069706	-0.36671	1
performance	2	6	-29.9563	-0.22567	0.058566	-0.4519	1
performance	3	4	15.77476	-0.16248	0.235069	0.140359	1
performance	3	5	2.869814	-0.11941	0.134277	0.045034	1
performance	3	6	-4.49049	-0.13377	0.108723	-0.0794	1

Parameter	Cond1	Cond2	RelDiff Cond1-Cond2	CI95_Low	CI95_High	Cohen's_dz	p_bonferroni
Performance	4	5	-11.1466	-0.26096	0.203232	-0.09559	1
performance	4	6	-17.504	-0.24767	0.150029	-0.18872	1
performance	5	6	-7.15497	-0.20705	0.167136	-0.08199	1
